# Draft Genome Sequence of the Filamentous Cyanobacterium *Leptolyngbya* sp. Strain Heron Island J, Exhibiting Chromatic Acclimation

**DOI:** 10.1128/genomeA.01166-13

**Published:** 2014-02-06

**Authors:** Robin Paul, Robert E. Jinkerson, Kristina Buss, Jason Steel, Remus Mohr, Wolfgang R. Hess, Min Chen, Petra Fromme

**Affiliations:** a Department of Chemistry and Biochemistry, Arizona State University, Tempe, Arizona, USA; b Department of Chemistry and Geochemistry, Colorado School of Mines, Golden, Colorado, USA; c Biodesign Institute, Arizona State University, Tempe, Arizona, USA; d Genetics and Experimental Bioinformatics, University of Freiburg, Institute of Biology III, Freiburg, Germany; e School of Biological Sciences, the University of Sydney, Sydney, NSW, Australia

## Abstract

*Leptolyngbya* sp. strain Heron Island is a cyanobacterium exhibiting chromatic acclimation. However, this strain has strong interactions with other bacteria, making it impossible to obtain axenic cultures for sequencing. A protocol involving an analysis of tetranucleotide frequencies, G+C content, and BLAST searches has been described for separating the cyanobacterial scaffolds from those of its cooccurring bacteria.

## GENOME ANNOUNCEMENT

*Leptolyngbya* sp. strain Heron Island is a cyanobacterium classified in section III, a section that consists of filamentous cyanobacteria that grow in a single plane (1). This cyanobacterium was isolated from Heron Island, Australia, in 2009. *Leptolyngbya* sp. Heron Island exhibits chromatic acclimation, a phenomenon in which the composition of the light-harvesting complex phycobilisome (PBS) is modified with changes in light conditions (2–4).

DNA was isolated from *Leptolyngbya* sp. Heron Island using the Qiagen DNeasy plant minikit. The genomic DNA was broken into fragments by sonication for 5 min. The genome of *Leptolyngbya* sp. Heron Island was sequenced using an Illumina HiSeq 2000, applying the short-insert paired-end sequencing protocol (100-bp insert size) to a depth of coverage of 100×. These reads were *de novo* assembled using the ABySS (version 1.3.6) software (5). *Leptolyngbya* sp. Heron Island cultures cannot be grown axenically; therefore, the genomic assembly harbors sequences from other heterotrophic bacteria that are associated with this cyanobacterium. Several approaches were used to detect and separate any heterotrophic bacterial sequences or chimeras from the *Leptolyngbya* sp. Heron Island genome assembly. They include a G+C percentage analysis, a tetranucleotide frequency analysis, comparison to a reference cyanobacterial genome, and an analysis of gene annotations. The initial G+C percentage analysis of the assembled scaffolds indicated that 3 to 4 contaminating genomes are likely present. The first step consisted of devising a BLAST algorithm that selected only those scaffolds that contained a gene that matched some gene in *Leptolyngbya* sp. strain PCC 7376. The tetranucleotide frequencies (6) of the BLAST positive scaffolds were calculated using TETRA (7), followed by principal component analysis (8) using Biopython (9) of the resultant matrix obtained from TETRA. Finally, the scaffolds were separated on the basis of their G+C percentage to yield scaffolds that contained only cyanobacterial genes.

Genome annotation was carried out using GeneMark S (10) and the NCBI Prokaryotic Genomes Annotation Pipeline (PGAP) 2.0. The resulting gene models were validated against the NCBI nonredundant protein database by BLASTp, which yielded maximum sequence homology with cyanobacterial genes.

The final assembly of *Leptolyngbya* sp. Heron Island contains 119 scaffolds, with an N_50_ scaffold length of 103,122 bp and an overall assembly size of 8.06 Mb, making it one of the biggest genome sequences among cyanobacteria (11). The annotation includes 7,223 estimated protein-coding sequences, 55 tRNAs, and 9 clustered regularly interspaced short palindromic repeat (CRISPR) arrays. The latter component suggests an amazing capability to deal with attacking bacteriophages, as CRISPR arrays are a key defense mechanism against invading DNA (12), and it is consistent with a report that subsection III and IV cyanobacteria tend to have a higher number of CRISPR loci and of repeat-spacer units (13). The availability of the sequences of the phycobilisome genes in *Leptolyngbya* sp. Heron Island will help in the refinement of phycoerythrin and phycocyanin crystal structure obtained from this organism. Eventually, with the detailed phycobilisome-encoding genes, the genome information will also help in studying chromatic acclimation in this newly isolated cyanobacterium.

### Nucleotide sequence accession number.

The draft genome sequence of *Leptolyngbya* sp. strain Heron Island J has been deposited at DDBJ/EMBL/GenBank under the accession no. AWNH00000000.1. The version described in this paper is the first version.
